# The role of Spectral Domain Optical Coherence Tomography in monitoring uncontrolled hypertensive type 2 diabetic patients

**Published:** 2014

**Authors:** D Stana, R Iancu, C Leasu, V Popescu, A Dumitrescu, S Gradinaru

**Affiliations:** * Ophthalmology Clinic, University Emergency Hospital Bucharest, Bucharest, Romania; ** Ophthalmology Department, ”Carol Davila” University of Medicine and Pharmacy, Bucharest, Romania; *** Anatomy Department, ”Carol Davila” University of Medicine and Pharmacy, Bucharest, Romania

**Keywords:** spectral domain optical coherence tomography, hypertensive, type 2 diabetic patients

## Abstract

**Rationale:** The pathogenesis of diabetic retinopathy is multifactorial, and a range of hyperglycemia-linked pathways has been implicated in the initiation and progression of this condition. All the cells in the retina are affected by the diabetic milieu, and in view of such disease and tissue complexity, it is unlikely that any single process is solely responsible for the retinal pathophysiology. Dyslipidemia is considered a trigger to rapid worsening of the condition and its treatment is becoming a part of normal diabetes treatment. Nevertheless, as establishing causal mechanisms and related conditions remain an important research goal, also the means to follow up the impact on the retina and other ocular tissues are as important.

**Objective:** this retrospective study shows the progression of diabetic macular edema (DME) in patients with dyslipidemia related to poor glycemic and blood control in subjects with existing DME by measuring the total macular volume (TMV) and thickness through the spectral domain optical coherence tomography (SD- OCT).

**Methods and results:** 30 uncontrolled cases of type 2 diabetes that were measured monthly by SD- OCT through a period of 3 months with correlation to the degree of dyslipidemia and hyperglycemia, were analyzed.

**Conclusion:** The role of OCT in monitoring the progression of DME in patients with uncontrolled type 2 diabetes is essential and the collaboration between the ophthalmologist and endocrinologist is essential to monitor the course of disease in uncontrolled patients.

## Introduction

The worldwide incidence of diabetes is believed to rise dramatically from 171 million people to an estimated 366 million in 2030 [**1**].The frequency of Type 1 diabetes is low relative to Type 2 diabetes, which accounts for approximately 90% of diabetes worldwide [**2**]. One of the major causes of blindness among the population at working age is diabetic macular edema (DME) and risk factors for diabetic retinopathy are sometimes difficult to manage. Almost 30 years ago, the Early Treatment Diabetic Retinopathy Study (ETDRS) found that DME, diagnosed by means of stereoscopic fundus photography, has led to moderate visual loss in one of four people within three years.

At present, the best advice for diabetic patients is to maintain their glycemic control, indicated by HbA1c, as close to normal limits as possible (normal levels considered under 6.4%). This was proven by DCCT (Diabetes Control and Complications Trial) for Type 1 diabetes conducted from 1983 to 1993 [**3**], although the benefits of so-called “tight” glycemic control took several years to manifest. Similarly, for Type 2 diabetes, UKPDS (UK Prospective Diabetes Study) demonstrated that maintaining near normal glycaemia could significantly protect against the progression of diabetic retinopathy [**4**].

Hyperglycemia was investigated in many studies on diabetic retinopathy, although it is clear that dyslipidemia is also an important risk factor and that regulation of blood lipids should be considered as an effective means to offset the progression of this complication [**5**]. The seminal Hoorn Study identified dyslipidemia as a clear additional risk factor for diabetic patients [**6**]. This is also shown by clinical trials such as FIELD (Fenofibrate Intervention and Event Lowering in Diabetes) [**7**] and ACCORD (Action to Control Cardiovascular Risk in Diabetes) [**8**], which evaluated the role of fenofibrate and simvastatin + fenofibrate respectively.

UK Prospective Diabetic Study highlighted the importance of BP (blood pressure) as well as hyperglycemia in diabetic retinopathy [**4**,**9**]. More recent studies, such as the META-EYE (Meta-Analysis for Eye Disease) study, have demonstrated a prevalence of diabetic retinopathy among people with normal BP of 5.5% compared with 10.6% in those with hypertension (BP >140/ 90mmHg or already on antihypertensive medication) [**10**]. It is now established that patients with diabetes should avoid systemic hypertension, which exacerbates initiation and progression of retinopathy [**11**]. Patients with untreated diabetes mellitus and insulin deficiency commonly have hypertriglyceridemia; this condition occurring more frequently in type 2 than in type 1 diabetes mellitus. Appropriate diabetes management reduces triglyceride levels. Mild hypertriglyceridemia, typically seen in treated type 2 diabetes, is probably related to the presence of central obesity and insulin resistance [**12**].

Diabetic macular edema (DME) is a thickening of the central retina, or the macula, and is associated with long-term visual loss in people with diabetic retinopathy.

Optical coherence tomography (OCT) is based on optical reflectivity and is able to image retinal thickness and structure producing cross-sectional and three-dimensional images of the central retina. It is already widely used because it provides objective and quantitative assessment of macular edema unlike the subjectivity of fundus biomicroscopic assessment, which is routinely used by ophthalmologists instead of photography. Optical coherence tomography is also used for quantitative follow up of the effects of treatment of DME [**13**].

## Material and method

Based on our recordings, we analyzed 30 patients (60 eyes) with uncontrolled type 2 diabetes and DME that were referred by endocrinologist for regular fundus exam. At admission, all patients had blood samples for glycaemia, HbA1c, total cholesterol levels, triglycerides, HDL and LDL cholesterol levels, blood pressure measurements and OCT performed after normal ocular examination. The patients were monitored for 3 months by the endocrinologist and by the ophthalmologist in order to obtain better diabetes and also retinopathy control. The study group consisted of 15 men and 15 women (mean age 52 years for women and 50 for men) who were admitted in the University Emergency Hospital Bucharest between January 2014 and March 2014. At hospital admission, all the patients signed an informed consent. All the patients were treated with oral antidiabetic medication started by the endocrinologist for more than 4 months prior to the OCT exam.

Inclusion criteria were considered: 

• blood pressure (BP)>170/ 100 mm Hg,

• glycaemia >150 mg/ ml 

• HBA1c >6.9%

• Total cholesterol levels >200 mg/ ml

• DME on OCT exam

Exclusion criteria were the following:

• Patient without diabetic treatment

• BO < 170/ 100 mm Hg

• Absence of DME

• Glycaemia <150 mg/ dl

• HbA1c <6.4%

The OCT measurements consisted in total macular volume (TMV) and central macular thickness in all patients for 3 months (3 exams in total) related to the BP, lipids profile and glycaemia.

## Results

The patients were divided in two groups: group A with stable values (considered with less than 10% variation from baseline) for glycaemia, BP and lipids profile and group B with more than 20% variation from baseline (considered the first exam). The groups were selected to match from the sex and age variables.

For group A, with a mean BP of 180/ 100 mm hg, glycaemia >160 mg/ ml , HbA1c >7%, total cholesterol levels >220 mg/ dl, triglyceride levels >180 mg/ dl and LDL levels > 60 mg/ dl, the TMV at baseline was 10.28 mm3 and the central macular thickness was 283,46 microns, and, at 3 months, the TMV was 10.9 mm3 and the central macular thickness was 303.26 microns.

In group B, with a mean BP of 220/ 100 mm hg, glycaemia >180 mg/ dl, HbA1c >7.4%, total cholesterol levels >220 mg/ dl, triglyceride levels >200 mg/ dl and LDL levels >160 mg/ dl, the TMV at baseline was 11.25 mm3 and the central macular thickness was 318,28 microns, and, at 3 months, the TMV was 13.2 mm3 and the central macular thickness was 363.26 microns.

## Discussion

The most commonly used grading system in the clinical and epidemiological studies of diabetic retinopathy is the ETDRS (Early Treatment of Diabetic Retinopathy Study) scale [**14**], which relies upon a number of photographically detectable microvascular lesions as indicators of disease progression. Levels of non-proliferative diabetic retinopathy are characterized by the number and severity of microaneurysms, dot and blot haemorrhages, cotton wool spots (nerve fiber layer infarcts), venous abnormalities (beading and looping) and IRMAs (intraretinal microvascular anomalies), which are large-caliber shunt vessels within non-perfused regions of the capillary bed. Proliferative diabetic retinopathy involves the formation of new blood vessels and is graded according to the extent and location of new vessels at the optic disc or elsewhere on the fundus and the presence or absence of vitreous haemorrhage. Microaneurysms are hallmark lesions of diabetic retinopathy. Ophthalmoscopically, microaneurysms may appear as dark red or white spots in the fundus, whereas fluorescein angiography reveals perfused microaneurysms as discrete hyperfluorescent spots [**15**]. 

The extent of diabetic macular edema and its variability related to the adherence to treatment, blood pressure and dyslipidemia control are finer parameters to be observed [**16**] and OCT represents the mean to achieve this information. Variability of parameters in this uncontrolled groups accounted for the increase of DME in a 3 months follow up and OCT should be considered a normal tool for screening uncontrolled diabetic patients. Endocrinologists should refer the uncontrolled diabetic patient for more regular check-ups that include OCT exam also in order to find the best therapeutic scheme that controls the progression of the disease.

## Conclusion

Long-term management of retinopathy in the ever-expanding diabetic patient population involves precise regulation of glycemic, vasotensive, and lipidemic profiles, most effectively in combination with drugs that ameliorate an array of biochemical and metabolic abnormalities. The synergy between the ophthalmologist’s work and the endocrinologist’s is crucial, as special imaging tools are needed permanently in the attempt to control the diabetic patients regularly.
